# Antiviral Activity of 1-Deoxynojirimycin Extracts of Mulberry Leaves Against Porcine Epidemic Diarrhea Virus

**DOI:** 10.3390/ani15091207

**Published:** 2025-04-23

**Authors:** Yiwei Sun, Liyan Wang, Keke Ma, Manman Shen, Jiying Liu, Yujuan Zhang, Liumei Sun

**Affiliations:** 1 Jiangsu Key Laboratory of Sericultural and Animal Biotechnology, School of Biotechnology, Jiangsu University of Science and Technology, Zhenjiang 212100, China; 221211802111@stu.just.edu.cn (Y.S.); stef1214@163.com (L.W.); kekema18@163.com (K.M.); mms@just.edu.cn (M.S.); liujiying@just.edu.cn (J.L.); zhangyujuan@just.edu.cn (Y.Z.); 2 Key Laboratory of Silkworm and Mulberry Genetic Improvement, Ministry of Agriculture and Rural Affairs, Sericultural Scientific Research Center, Chinese Academy of Agricultural Sciences, Zhenjiang 212100, China

**Keywords:** PEDV, 1-deoxynojirimycin (DNJ), antiviral activity

## Abstract

Porcine epidemic diarrhea virus (PEDV), a highly infectious alphacoronavirus, has resulted in substantial economic losses within the global swine industry. Existing vaccines and therapeutic agents have proven inadequate in effectively preventing and controlling PEDV. Natural compounds offer distinct advantages in antiviral research due to their abundant availability, diverse biological activities, and low toxicity. In this study, the antiviral properties of the naturally occurring alkaloid 1-deoxynojirimycin (DNJ) against PEDV were examined. The CC_50_ of DNJ was determined to be 912.5 μM through experimental analysis on Vero-E6 cells. DNJ demonstrated an inhibitory effect on PEDV activity, with a 50% inhibitory concentration (IC_50_) of 57.76 μM. The compound primarily inhibited PEDV proliferation during the viral life cycle stages of attachment and replication. Moreover, DNJ mitigated the production of reactive oxygen species (ROS) and inflammation associated with PEDV infection. Computational docking predictions suggest that the viral non-structural proteins include Nsp12, Nsp14, and Nsp16 may serve as potential targets for DNJ. Consequently, DNJ represents a promising candidate for the development of novel therapeutic agents against PEDV.

## Simple Summary

Porcine epidemic diarrhea virus (PEDV) represents a significant pathogenic threat within the swine industry, characterized by its high infectivity and substantial economic impact globally. Existing vaccines and therapeutic interventions have demonstrated limited efficacy in the clinical prevention and management of PEDV. Consequently, the identification of potent natural compounds with anti-PEDV properties is a critical strategy for addressing this challenge. This study investigates the anti-PEDV effects of 1-deoxynojirimycin (DNJ), derived from mulberry leaf extracts. Our findings reveal that DNJ effectively inhibits PEDV proliferation, primarily during the attachment and replication phases within Vero cells. Additionally, DNJ was shown to reduce reactive oxygen species (ROS) levels, thereby inhibiting PEDV replication and mitigating the inflammatory response associated with PEDV infection. Autodock predictions further suggest that the viral non-structural proteins may serve as potential targets for DNJ. These results indicate that DNJ holds promise as a resource for the development of novel therapeutic agents against PEDV.

## 1. Introduction

Porcine epidemic diarrhea virus (PEDV) is one of the important pathogens causing porcine diarrhea disease. PEDV infection leads to nearly 100% mortality in suckling piglets [1]. The global spread of PEDV has caused significant economic losses and public health risks to the pig industry. PEDV belongs to the Coronaviridae family, with a single-stranded sense RNA genome of about 28 kb [2]. There are 7 open reading frames (ORF1a, ORF1b, spike (S) glycoprotein gene, ORF3 hypothetical protein gene, envelope (E) protein gene, membrane (M) glycoprotein gene and nucleocapsid (N) protein gene between the 5′ untranslated region UTR and the 3′ untranslated region UTR [3]. The S protein is the main envelope glycoprotein responsible for virus attachment, receptor binding, cell membrane fusion, and entry. The N protein binds to the viral genomic RNA to form a helical nucleocapsid, which plays an important role in the release of viral particles from the cell. The virus enters the cell and releases its genome through membrane fusion or phagocytosis of receptor cells. Its life cycle includes attachment, entry, replication, and release. Firstly, PEDV recognizes the receptors of host cells and adsorbs onto the cellular surface [4]. Then, the virus enters the cell via clathrin-, caveolae-, and lipid raft-mediated endocytosis and traffics through the endo–lysosome pathway [5]. After entering the cell, the virion replicates in the cytoplasm, including the release, replication, and expression of the genome and the assembly of the virus. Finally, the virion is secreted from the host cell by cytosolic exocytosis.

At present, traditional vaccines cannot provide effective protection against PEDV mutant strains, it is particularly urgent to find potential antiviral drugs. A large number of studies have shown that extracts of traditional Chinese medicine can affect one or more stages of the PEDV life cycle, reducing the expression of viral proteins and inhibiting viral infection. Traditional Chinese medicine documents mulberry as a rich source of bioactive compounds, particularly alkaloids such as 1-deoxynojirimycin (DNJ). DNJ is a characteristic component of polyhydroxy alkaloids in mulberry leaves, which has anti-hyperglycemia, anti-obesity, anti-virus, and anti-tumor activities [6,7]. So far, dozens of DNJ derivatives have been synthesized, some of which have been used in clinical treatment of certain diseases. In addition, current studies have found that DNJ and its derivatives hinder the proliferation of HIV in human cells by affecting the adhesion of sugar molecules to the virus shell, thereby potentially avoiding HIV infection in the human body [8,9]. The derivatives of DNJ inhibit hepatitis B virus (HBV) viral activity by affecting the function of glycoproteins, which in turn inhibits cell fusion and syncytium formation [10,11]. Similar mechanisms, such as those of BDBV and dengue virus (DENV), suggest that DNJ also has antiviral activity [12]. Reports also exist about influenza viruses that emphasize the antiviral properties of MON-DNJ against H1N1, as observed in mouse models [13]. However, in relation to coronaviruses, there has been only one study examining the effectiveness of DNJ derivatives against SARS-CoV-2 in vitro [14]. No further research has been performed on the efficacy of DNJ concerning coronaviruses. Whether DNJ has anti-PEDV activity remains to be studied.

In addition, DNJ has anti-inflammatory and antioxidant activities, DNJ treatment reduced the levels of interleukin-6 (IL-6), tumor necrosis factor-α (TNF-α), and lipopolysaccharide (LPS) associated with insulin resistance [15,16,17]. DNJ mitigates oxidative stress through free radical scavenging and enhanced superoxide dismutase (SOD) activity, catalase (CAT), glutathione peroxidase (GSH-Px) activity, and reducing malondialdehyde (MDA) activity [17,18]. DNJ treatment can alleviate angina pectoris in patients with coronary heart disease (CHD) and blood stasis syndrome (BSS), increase SOD levels, and improve symptoms of shortness of breath and emotional uneasiness [19]. Some scholars have found that DNJ treatment in gastric ulcer (GU) mice increases the activity of SOD, CAT, and GSH, while decreasing MDA levels, thereby alleviating oxidative stress in GU mice [20]. Oxidative stress interacts with inflammatory response, and oxidative stress can induce inflammatory response. Inflammation will release inflammatory factors. Research showed a decrease in the content of inflammatory factors such as TNF-α, IL-1β, and IL-6 in the liver of mice, and found that the treatment of mulberry leaf alkaloids can alleviate the inflammatory response induced by alcohol exposure [21]. Virus infections typically induce inflammation, which can exacerbate the inflammatory response. The effect of DNJ processing on pro-inflammatory factors and various oxidative indicators in cells infected with PEDV warrants in-depth investigation.

This study investigated the function of DNJ in PEDV replication. It demonstrated that PEDV mainly exerts its antiviral function during the attachment and replication stage of virus life cycle. Additionally, molecular docking studies indicated that DNJ demonstrates a robust binding affinity to various viral non-structural proteins. It also showed that DNJ can reduce reactive oxygen species (ROS) to inhibit PEDV replication and decrease the inflammatory response induced by PEDV infection in Vero cells. In summary, these results established that DNJ inhibits PEDV infection by decreasing ROS generation.

## 2. Materials and Methods

### 2.1. Cells, Viruses, and Antibodies

Vero-E6 cells were cultured in Dulbecco’s modified Eagle’s medium (DMEM) (Gibco, C11995500BT, New York, NY, USA) supplemented with 10% fetal bovine serum (FBS) (VivaCell, 040642, Shanghai, China) and 1% penicillin–streptomycin (Psaitong, U31-301C, Beijing, China) and incubated at 37 °C with 5% CO_2_. The PEDV strain SQ2014 (GenBank accession No. KP728470) and Rabbit anti-PEDV N protein polyclonal antibodies were kindly provided by Professor Qian Yingjuan (Nanjing Agricultural University) [22]. β-Actin Rabbit polyclonal antibodies were purchased from Proteintech (20536-1-AP, Nanjing, China). HRP Goat Anti-Rabbit IgG antibody was procured from ABclonal (NO. AS014, Wuhan, China).

### 2.2. Cytotoxicity Assay

The Cell Counting kit-8 (CCK8) (Beyotime, C0038, Shanghai, China) was used to measure cell viability according to the instructions mentioned. Cells were seeded into 96-well plates at a density of 1.5 × 10^4^ cells/well. Following a 12 h incubation at 37 °C, the medium was aspirated, and cells were washed three times with PBS (Servicebio, G4202-500 mL, Wuhan, China). The complete medium containing different concentrations of DNJ was added to the 96-well plate, and three duplicate wells were set for each drug concentration. After a 16 h incubation, the medium was removed, and the cells were rinsed with PBS three times. Subsequently, a complete medium containing 10% CCK-8 was added to each well of the 96-well plate under dark conditions, and the culture plate was placed in the incubator (Thermo, Shanghai, China) for 2 h. After gentle shaking for 5 min, each well’s optical density (OD) value was measured at the wavelength of 450 nm with a microplate reader (Bio-Walker, EPOCH2NS, Nanjing, China). The drug’s inhibition rate was determined by employing the formula: Inhibition rate (%) = [(As − Ab)/(Ac − Ab)] × 100. Here, As represents the absorbance recorded from the test well containing cells, culture medium, CCK-8, and the compound under investigation. Ab signifies the absorbance of the blank well, which includes solely the culture medium and CCK-8, while Ac denotes the absorbance from the control well, consisting of cells, culture medium, CCK-8, and DMSO.

### 2.3. Determination of Half Virus Inhibition Rate

In order to determine the antiviral activity of DNJ against PEDV infection, Vero cells were fixed with 4% paraformaldehyde for 30 min, rinsed with Glycine-PBS solution three times and then rinsed with PBS, and 3% bovine serum albumin (BSA) was used to block non-specific binding. After blocking for one hour, the cells were incubated with Rabbit anti-PEDV N protein polyclonal antibodies at 37 °C for one hour. The cells were washed three times with PBS, and then incubated with the HRP Goat Anti-Rabbit IgG antibody for 60 min at 37 °C. After washing, the TMB color development solution was used, and the color development was performed at room temperature for 30 min in the dark, and 2% H_2_SO_4_ solution was added to each well to terminate the color development. Finally, after slowly shaking on the shaker for 5 min, the absorbance at 450 nm was measured using a microplate reader, and the inhibition rate of the drug was calculated using the following formula: The inhibition rate of the drug (%) = [(As − Ab)/(Ac − Ab)] × 100, where As represents the absorbance of the test hole (the absorbance of the virus hole infected by cells treated with different concentrations of drugs); Ab represents the absorbance of the blank hole (the absorbance of the hole containing only the chromogenic solution and the termination solution); Ac represents the absorbance of the control hole (the absorbance of the virus hole infected by cells treated with DMSO).

### 2.4. Quantitative Real-Time PCR

Total RNA was extracted from Vero cells using TRIzol Reagent (Vazyme, R401-01-AA, Nanjing, China) and reverse transcribed into cDNA using Evo M-MLV RT Mix Kit with gDNA Clean for qPCR Ver.2 (Accurate Biology, AG11728, Hunan, China) according to the manufacturer’s instruction, and gene expression was assessed by qRT-PCR using specific primers (Table 1). The samples were evaluated in triplicate using SYBR (Vazyme, Q312-02, Nanjing, China). The data were analyzed using the 2^−∆∆Ct^ method, and expression was normalized against GAPDH level.

### 2.5. Western Blotting

Cells in 6-well plates were washed thrice with PBS and then SDS-PAGE Sample Loading Buffer (2×) was added to the samples. After gentle shaking for 5 min, the samples were collected into a 1.5 mL centrifuge tube for ultrasonic cell disruption. Cooled samples were ultrasonicated for 3 min per the power of 120 w. Subsequently, the samples were incubated at 100 °C for 10 min and centrifuged at 10,000× rpm for 5 min. The supernatant was loaded onto SDS-PAGE gels (ACE, ET15420Ge1, Nanjing, China) and subjected to electrophoresis, and separated proteins were transferred onto 0.45 µM TM-NC-R-45 membranes (LABSELECT, TMNC-R-45, Beijing, China). In the following step, the membranes were incubated with 3% skim milk (diluting in PBS buffer containing 0.1% Tween-20, PBST) for 1 h at room temperature, followed by PBST washes three times. The membranes were then incubated in a solution containing 0.01% indicated antibodies at 4 °C overnight. The membranes were rinsed with PBST three times and then kept in a solution containing 0.3% HRP Goat Anti-Rabbit IgG antibody for 4 h at 4 °C. Finally, the membranes were washed with PBST three times and subjected to Super ECL Detection Reagent ECL (YEASEN, 36208ES60, Shanghai, China). These images were captured through a chemiluminescence detector (Clinx, ChemiScope 6100, Shanghai, China).

### 2.6. The Experiment of DNJ Inhibiting the Life Cycle of PEDV

In inactivation assay, Vero-E6 cells (1 × 10^6^ cells per well) were first cultured in a 6-well plate at 37 °C for 12 h, followed by washing the cells three times with PBS. PEDV with MOI = 0.1 was mixed with DNJ (25, 50, 75, 100 µM) or DMSO, and the volume of the mixture was 1 mL. The mixture was placed in a 1.5 mL centrifuge tube and incubated at 37 °C for 1 h. After this period, the resulting mixture was diluted with serum-free medium, inoculated onto Vero-E6 cells, and incubated once more at 37 °C for an additional hour. Subsequently, PEDV maintenance medium (2% DMEM supplemented with 0.25% trypsin) containing DNJ was added to each well, and the cells were further cultured at 37 °C for 12 h. Then, the total protein content from the cell was collected for Western blot analysis.

In virus attachment assay, Vero-E6 cells (1 × 10^6^ cells per well) were first cultured in a 6-well plate at 37 °C for 12 h, then washed three times with PBS. Vero-E6 cells were incubated with DNJ at a concentration of 50 µM at both 37 °C and 4 °C for one hour consecutively. Afterward, the cells were infected with PEDV SQ2014 (MOI = 0.1) and co-incubated at 4 °C for another hour. The infected cells were washed three times using cold PBS, and total RNA from cells was obtained for RT-qPCR analysis.

In virus entry assay, Vero-E6 cells (1 × 10^6^ cells per well) were first cultured in a 6-well plate at 37 °C for 12 h, followed by washing the cells three times with PBS. Vero-E6 cells underwent pre-chilling at 4 °C for one hour before being infected with PEDV SQ2014 (MOI = 0.1) at the same temperature for one hour. Subsequently, DNJ (50 µM) was introduced, and the cells were incubated at 37 °C for two hours to promote the internalization of the virus. After conducting three washes with citrate buffer at pH 3, total RNA from cells were collected for RT-qPCR analysis.

In virus replication and release assay, Vero-E6 cells (1 × 10^6^ cells per well) were first cultured in a 6-well plate at 37 °C for 12 h and then washed three times with PBS. Vero-E6 cells were again pre-chilled at 4 °C for one hour, followed by PEDV SQ2014 infection (MOI = 0.1) at 4 °C for one hour. DNJ (50 µM) was added afterwards, and the cells were incubated for two hours at 37 °C to facilitate virus internalization. Following three rinses with citrate buffer at pH 3, Vero-E6 cells were cultured in maintenance medium (2% DMEM supplemented with 0.25% trypsin) containing DNJ (50 µM) at 37 °C for three hours. Total RNA from cells was collected for RT-PCR analysis. And virions from cells and maintenance medium were collected for viral plaque formation assay, respectively.

### 2.7. Plaque Formation Assay

The virus supernatant containing continuous dilution of PEDV was incubated with Vero cells in a six-well plate in an incubator at 37 °C for one hour, and the unbound virus was washed with PBS. Covering medium (2% low melting point agarose (BBI, A600015-0005, Shanghai, China) in DMEM containing 2% FBS) was added to each well, and the cells were cultured in an incubator (37 °C, 5% CO_2_) (Thermo, Shanghai, China) for 48–60 h. Plaque formation was observed after staining with 1% crystal violet (BBI, A600331-0025, Shanghai, China). The formation of virus plaques was photographed and imaged by chemiluminescence detector (Clinx, ChemiScope 6100, Shanghai, China).

### 2.8. Measurement of Anti-Oxidative Stress Activity

To detect total cellular reactive oxidative species (ROS), Vero cells (1 × 10^6^ cells) were infected with PEDV in the presence of DNJ; a fluorescence indicator DCFH-DA (2.5 µM) (Beyotime, S0033S, Shanghai, China) was incubated with Vero cells at 37 °C for 30 min. After incubation, cells were washed three times in serum-free cell culture medium and observed under an inverted fluorescence microscope (OLYMPUS, IX73, Tokyo, Japan). For GSH-Px and MDA measurements, cells were lysed by sonication and measured by a cellular glutathione peroxidase assay kit (Jiancheng, A005-1, Nanjing, China) and a lipid peroxidation MDA assay kit (Solarbio, BC0025, Beijing, China), following the manufacturers’ instructions, respectively.

### 2.9. Molecular Docking Simulation of the Binding of DNJ with the Protein Target of PEDV

The docking program utilized AutoDock Vina version 1.1.2, which involved the preprocessing, optimization, and minimization of both receptor and ligand molecules. A docking box was constructed to encompass the entire protein molecule, and a semi-flexible docking approach was employed during the simulation. Visualization of the selected docking results was performed using PyMOL 2.1 software to examine the interaction modes between the compounds and their respective target proteins. This analysis facilitates the exploration of specific interactions between the compounds and protein residues, including the formation of hydrogen bonds. Furthermore, inferences regarding the binding affinity of these compounds to the target protein were drawn based on their binding energy.

### 2.10. Statistical Analysis

The means and standard deviations of three independent experimental replicates are shown. The statistical analysis was accomplished by GraphPad Prism 6.0. Statistical differences between the two test groups were analyzed using Student’s *t*-test. Asterisks indicated significance as follows: * *p* < 0.05; ** *p* < 0.01; *** *p* < 0.001; **** *p* < 0.0001; ns, not significant.

## 3. Results

### 3.1. Evaluation of the Cytotoxicity of DNJ

**Figure 1:**
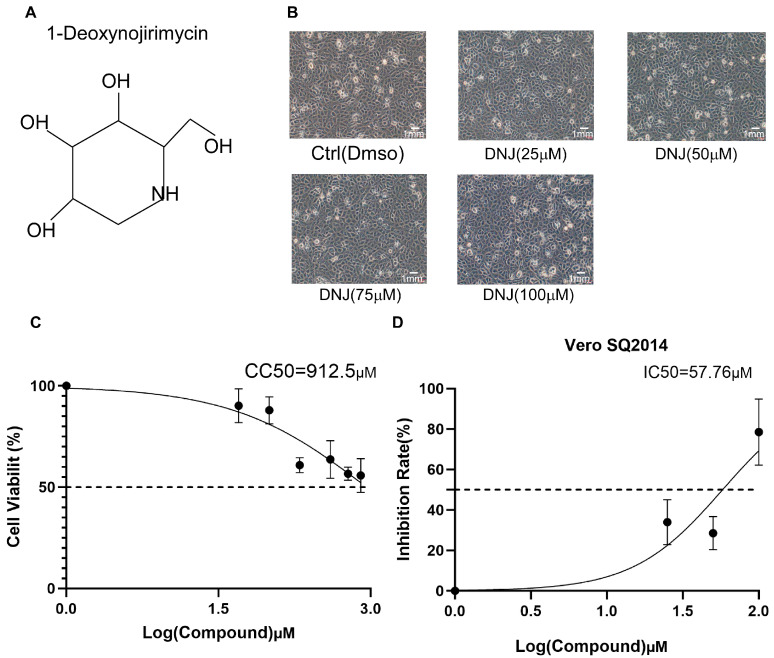
The chemical structure, CCK8, and IC_50_ of DNJ. (**A**) The chemical structure of DNJ. (**B**) Morphological changes were observed after the cells were treated with DNJ at different concentrations for 12 h using a fluorescence microscope under bright-field. (**C**) Vero E6 cells were treated with 0 μM, 50 μM, 100 μM, 200 μM, 400 μM, 600 μM, and 800 μM DNJ for 12 h, and then treated with CCK 8 for 2 h. The OD450 value was measured, and nonlinear regression analysis was performed to calculate the CC_50_ value. (**D**) The half maximal inhibitory concentration (IC_50_) was determined by cell ELISA, and the IC_50_ was calculated by nonlinear regression analysis.

Recent observations have highlighted DNJ’s hypoglycemic, hypolipidemic, anti-tumor, antiviral, and other pharmacological effects [23]. Its molecular structure is depicted in Figure 1A. The current literature indicates that DNJ derivatives can inhibit cell death induced by severe acute respiratory syndrome coronavirus type 2 in a dose-dependent manner, while concurrently reducing viral replication and spike protein levels [24,25]. Therefore, we hypothesized that DNJ may exert an inhibitory effect on PEDV replication. Consequently, we investigated the potential anti-PEDV activity of DNJ in Vero-E6 cells. Firstly, the CCK8 assay was used to evaluate the safe concentrations of DNJ on Vero cells. The CCK8 assay results (Figure 1B) and cellular state images (Figure 1C) illustrated that CC_50_ values (half cytotoxic concentration) of DNJ were observed on Vero-E6 cells. Then, the inhibitory effects of DNJ against PEDV were analyzed by the cell-based ELISA. The IC_50_ values of DNJ were 57.76 µM (Figure 1D). Further, the selectivity index (CC_50_/IC_50_) of DNJ was 15.798. Based on a comprehensive assessment, we selected DNJ concentration (less than 100 µM) with low cytotoxicity and an obvious effect on the Vero-E6 cells for further study.

### 3.2. Antiviral Activity of DNJ in Vero-E6 Cells

**Figure 2:**
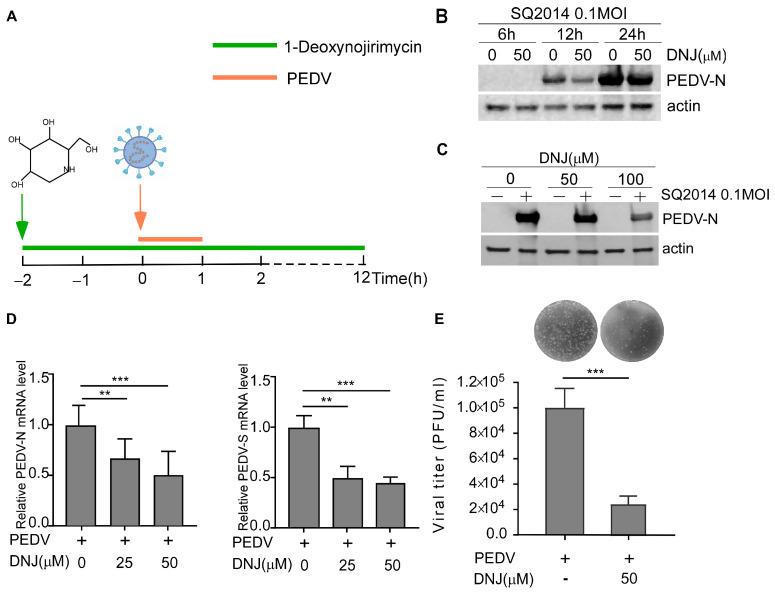
DNJ inhibits PEDV infection in Vero-E6 cells. (**A**) The overall design of Vero E6 cells infected and treated with DNJ (25 μM, 50 μM, 100 μM). The green line indicates drug treatment, and the orange line indicates PEDV infection. (**B**) Samples were collected at 6, 12, and 24 h post-infection with SQ2014 (MOI = 0.1), and the protein levels of PEDV-N and actin were detected by Western blot. (**C**) After 12 h of infection, the protein samples with different concentrations of DNJ were detected by Western blot. (**D**) Quantitative detection of PEDV S and N mRNA level by qRT-PCR. (**E**) The cells were collected 12 h after infection, and the virus titer of SQ2014 was detected by PFU. The means and standard deviations of three independent experimental replicates are shown. ** *p* < 0.01; *** *p* < 0.001.

To further explore the anti-PEDV effects of DNJ, Vero-E6 cells were infected with PEDV and treated with DNJ. The schematic diagram of DNJ treatment and PEDV infection process were shown in Figure 2A. As illustrated in Figure 2B (original data in Figure S1), the inhibitory effect of DNJ on PEDV was evaluated at various time points using Western blotting in Vero-E6 cells, specifically at 6, 12, and 24 h post-infection (h.p.i.) with PEDV at a multiplicity of infection (MOI) of 0.1. Notably, the inhibition of PEDV N by DNJ was particularly pronounced at 12 h post-infection. Western blot results showed that the expression of the PEDV N protein was diminished by DNJ in a manner dependent on dosage, showing the most pronounced inhibitory effect at a concentration of 100 µM (Figure 2C, original data in Figure S2). The qRT-PCR analysis revealed a significant reduction in PEDV N and S mRNA levels in DNJ-treated Vero cells (Figure 2D). Furthermore, the plaque formation assay confirmed that treatment with 50 µM DNJ significantly decreased the virus titer compared to the DMSO-treated group (Figure 2E). These findings demonstrate that DNJ possesses anti-PEDV properties and markedly suppresses PEDV infection as the concentration of the drug increases.

### 3.3. Effect of DNJ on Different Stages of PEDV Life Cycle

**Figure 3:**
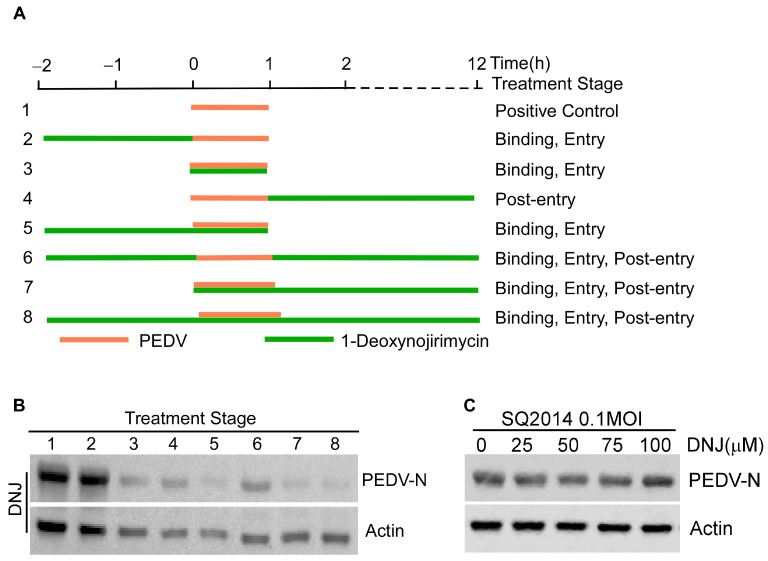
The inhibition stage of DNJ on PEDV. (**A**) This section describes the comprehensive approach for infecting Vero-E6 cells and administering DNJ at a concentration of 100 µM. The treatment procedure is divided into three separate phases: pre-treatment, co-treatment, and post-treatment. (**B**) In accordance with the treatment protocol depicted in (**A**), Western blot analysis was conducted to evaluate the protein levels of both the virus and actin. (**C**) DNJ directly targets PEDV SQ2014 experimental results.

**Figure 4:**
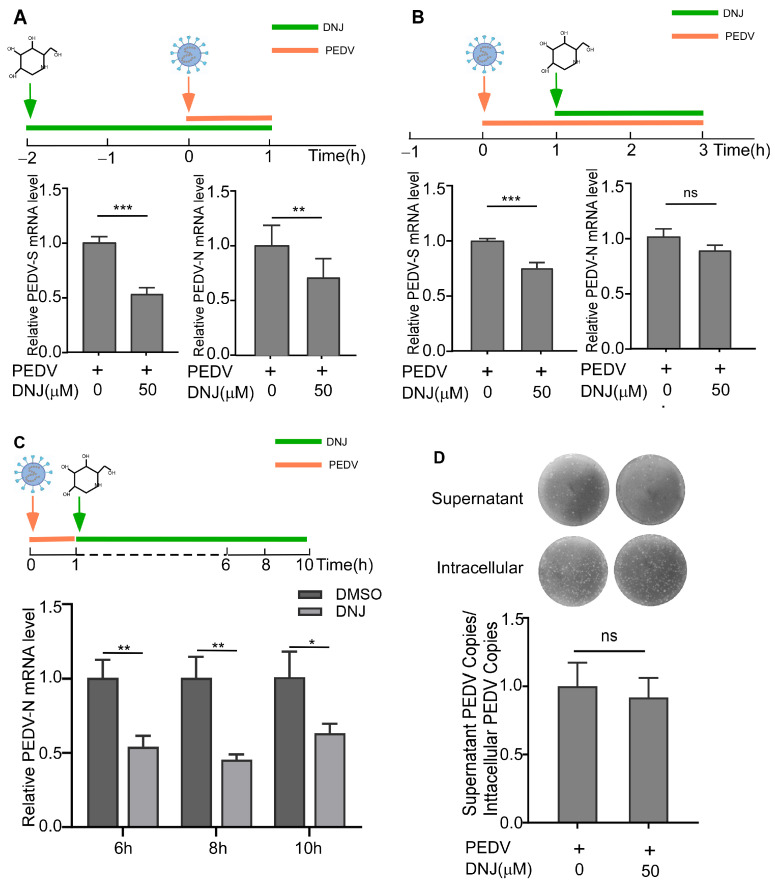
Effects of DNJ on the attachment, entry, replication, and release of PEDV. (**A**) The experimental design of DNJ and PEDV infection cycle stage treatment of Vero E6 cells. The green line segment indicates drug treatment, and the orange line segment indicates PEDV infection. Virus attachment experiment: the mRNA levels of PEDV S and N were detected by qRT-PCR after 1 h of infection with PEDV in DNJ-treated Vero E6 cells. (**B**) Virus entry experiment: PEDV S and N mRNA levels were detected by qRT-PCR 2 h after PEDV infection in Vero E6 cells treated with DNJ. (**C**) Virus replication experiment: Vero E6 cells were treated with DNJ within 6, 8, and 10 h post-infection, and the levels of PEDV N mRNA were detected by qRT-PCR. (**D**) Virus release assay: cells and supernatants were collected after virus infection, and PEDV titers in cells and supernatants were detected by PFU. The means and standard deviations of three independent experimental replicates are shown. * *p* < 0.05; ** *p* < 0.01; *** *p* < 0.001; ns, not significant.

The stages of the PEDV life cycle that were affected by DNJ were assessed using a time-of-addition assay (TOA). This experiment was categorized into eight groups according to the timing of treatment (Figure 3A). Results from Western blot analysis revealed that treatment during and following infection resulted in decreased levels of the viral protein PEDV-N. Remarkably, the largest reduction in PEDV-N levels occurred when DNJ was given during the course of the viral infection (Figure 3B, original data in Figure S3). To study the mechanism of DNJ’s anti-PEDV activity, we first examined whether DNJ could directly target PEDV particles using a direct virus-targeting assay. The results of the experiments showed no substantial difference in the expression levels of the viral protein PEDV-N between the experimental and control groups (Figure 3C, original data in Figure S4), implying that DNJ does not directly interact with viral particles to prevent PEDV infection. Then, we investigated DNJ effect on different stages of PEDV life cycle. The attachment, internalization, replication stage was measured by the relative amounts of PEDV S and N mRNA and β-actin using RT-qPCR. The release stage was measured by PFU analysis. Attachment assay results demonstrated that in comparison to the DMSO-treated control group, 50 µM DNJ-treated group led to a 47% and 30% decrease in the mRNA levels of PEDV S and N, respectively (Figure 4A). Internalization assay results showed that the levels of PEDV S mRNA were significantly decreased, while the levels of PEDV N mRNA were only mildly decreased (Figure 4B). The results indicated that cells collected at various time points post-infection were evaluated for viral replication, with DNJ significantly reducing the mRNA levels of PEDV N (Figure 4C). The release assay results showed that DNJ did not significantly impede the release of PEDV (Figure 4D). Overall, these findings imply that DNJ hampers PEDV replication, exhibiting the strongest inhibitory impact during the attachment phase, while other stages of the viral life cycle display varying levels of inhibition.

### 3.4. DNJ Anti-PEDV Activity Is Related to Inhibition of ROS Production

**Figure 5:**
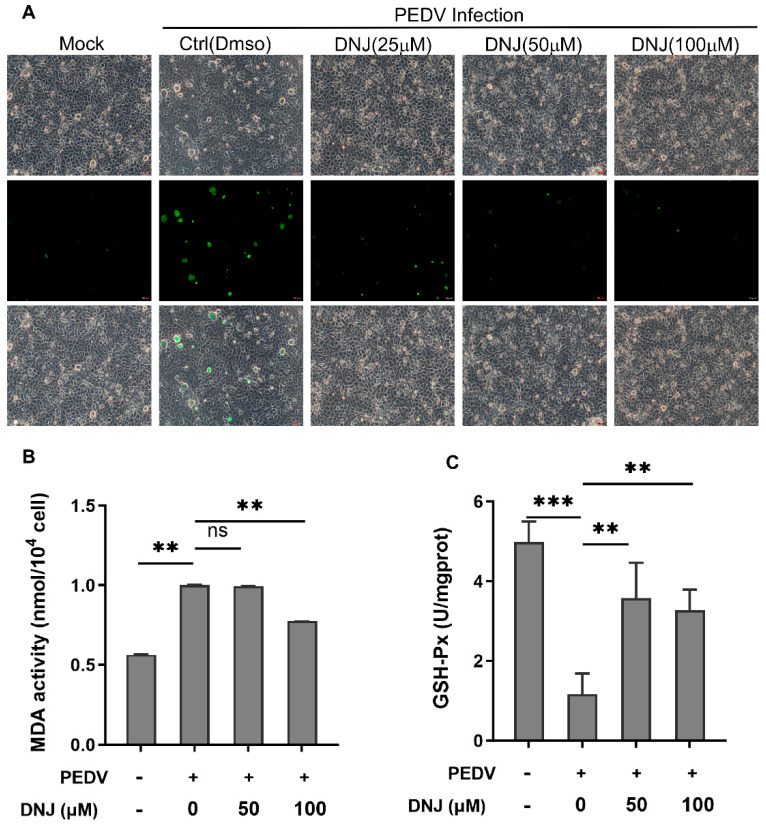
DNJ alleviates PEDV by inhibiting ROS production. (**A**) Vero cells were infected with PEDV (MOI = 0.1). After sufficient washing, the culture medium was replaced with different concentrations of DNJ to continue culturing. After 24 h, DCFH-DA was added and incubated for 40 min to detect cell ROS. (**B**) Production of MDA. (**C**) GSH-Px activity. The means and standard deviations of three independent experimental replicates are shown. ** *p* < 0.01; *** *p* < 0.001; ns, not significant. DNJ: 1-deoxynojirimycin (DNJ); PEDV: porcine epidemic diarrhea virus; ROS: reactive oxygen species.

Infection by PEDV can trigger both autophagy and apoptosis in Vero cells via signaling pathways that depend on reactive oxygen species (ROS) [26,27]. Research indicates that DNJ is capable of reducing ROS generation and mitigating the inflammatory response and apoptosis associated with septic cardiomyopathy [28]. To better understand the function of ROS in the antiviral effects of DNJ, we utilized the DCFH-DA probe for fluorescence detection of reactive oxygen species to monitor ROS level alterations in the drug-treated group versus the DMSO control during PEDV infection. As depicted in Figure 5A, DNJ significantly curtails ROS production in Vero cells infected by PEDV in a dose-dependent manner, indicating that the anti-PEDV effects of DNJ may involve the ROS pathway. Reactive oxygen species (ROS) are capable of prompting lipid peroxidation, which can consequently accelerate cell autophagy or apoptosis. Therefore, we employed malondialdehyde (MDA) as a biochemical marker to assess the impact of DNJ on lipid peroxidation levels. The findings represented in Figure 5B show that PEDV infection led to a marked increase in MDA production, whereas the inclusion of DNJ effectively mitigated this rise. In addition, total glutathione peroxidase (GSH-Px) plays a vital role in eliminating reactive oxygen species and products of peroxidation, and our studies revealed that DNJ was able to replenish GSH-Px activity that had been suppressed by PEDV infection (Figure 5C). These data suggested that DNJ anti-PEDV activity is related to inhibition of ROS production.

### 3.5. DNJ Alleviated the Inflammatory Cytokines Induced by PEDV Infection

**Figure 6:**
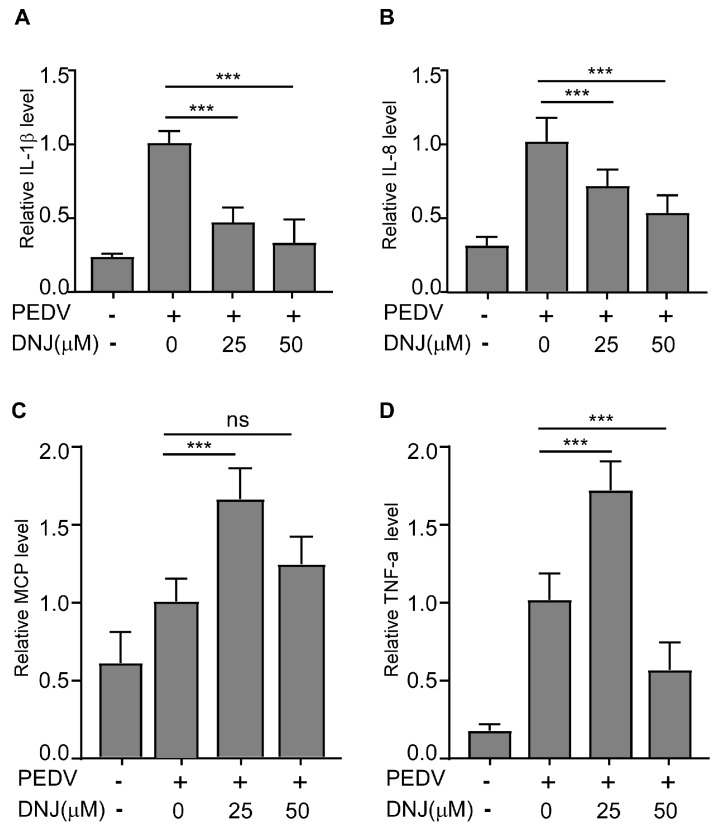
DNJ inhibited the expression of inflammatory cytokines and antioxidant indexes induced by PEDV infection. Vero E6 cells were infected with PEDV (MOI = 0.1) for 12 h with DNJ treatment. (**A**) The mRNA levels of IL-1β, (**B**) IL-8, (**C**) MCP, and (**D**) TNF-α were detected by RT-qPCR. The means and standard deviations of three independent experimental replicates are shown. *** *p* < 0.001; ns, not significant.

One of the cellular reactions to oxidative stress is inflammation [29]. Additionally, DNJ has been shown to boost both antioxidant and anti-inflammatory effects by lowering pro-inflammatory factor levels and influencing multiple signaling pathways [19,30]. Infection with PEDV induces inflammatory responses and increases the levels of inflammatory cytokines. To gain a deeper understanding of the mechanisms responsible for DNJ’s antiviral effects, the expression levels of inflammatory cytokines were evaluated through quantitative reverse transcription polymerase chain reaction (qRT-PCR). Findings revealed that DNJ notably reduced the mRNA levels of IL-1β and IL-8 that were elevated by PEDV (Figure 6A,B), indicating DNJ’s capacity to effectively suppress the inflammatory response triggered by the virus. Nevertheless, with rising concentrations of the drug, the mRNA levels of TNF-α and MCP did not achieve statistical significance when analyzed against the control group (Figure 6C,D). These data showed that DNJ alleviated the inflammatory cytokines induced by PEDV infection.

### 3.6. Molecular Docking to Predict Protein Targets of DNJ

**Figure 7:**
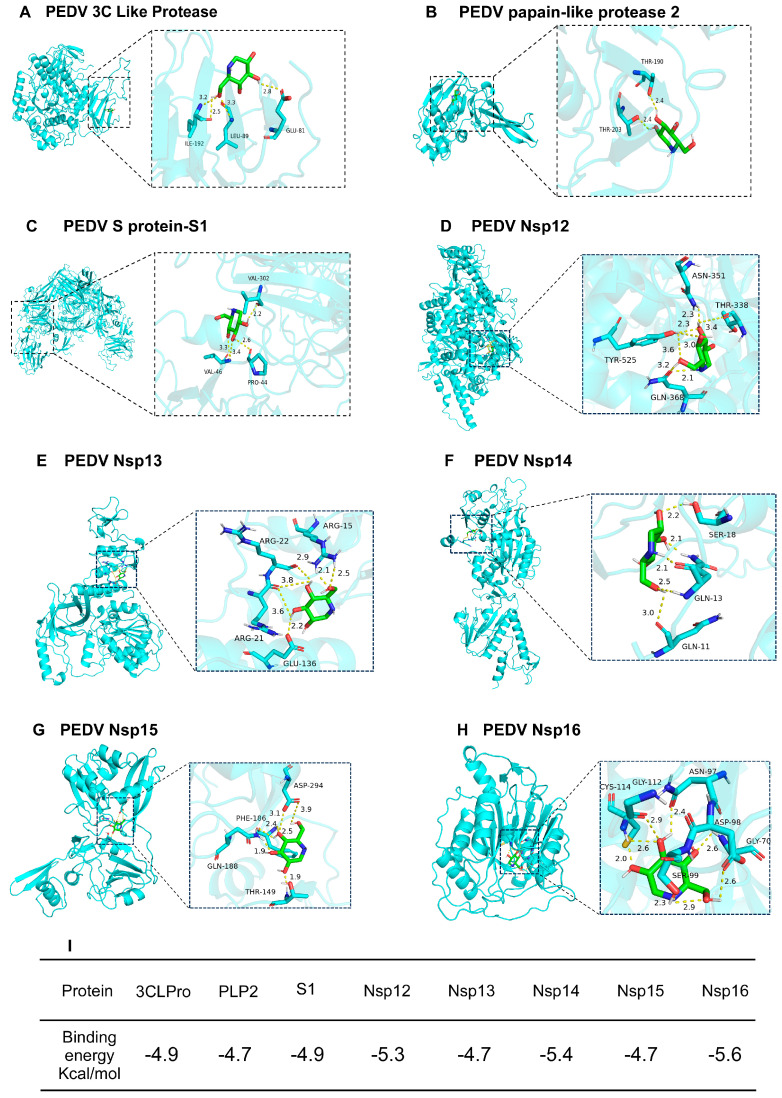
Molecular docking predicted the target of DNJ. The docking conformation of DNJ with (**A**) PEDV 3C Like Protease, (**B**) PEDV papain-like protease 2, (**C**) PEDV-S1, (**D**) PEDV Nsp12, (**E**) PEDV Nsp13, (**F**) PEDV Nsp14, (**G**) PEDV Nsp15, and (**H**) PEDV Nsp16. In this representation, the left diagram of each protein molecular complex is a 3D diagram of the structural conformation of the molecule and the protein, and the right diagram is a local interaction diagram of the hydrogen bond between the molecule and the protein. The green rod-like structure is a small molecule, and the orange rod-like structure is an amino acid residue that forms a hydrogen bond interaction in the protein. The amino acid name and site information are marked on it. The yellow dotted line represents the hydrogen bond, and the number next to the dotted line is the bond length. (**I**) The binding energy of the DNJ–protein complex, which was calculated using Autodock vina 1.1.2 software.

The research confirmed that DNJ has the potential to inhibit various protein targets of PEDV and shows notable binding affinity. We employed Autodock Vina 1.1.2 software for the molecular docking analysis (refer to Figure 7A–H) and partially visualized the docking results using PyMOL 2.1. The binding energy was generally observed to be lower than or around −5 kcal/mol, suggesting that the docking results are relatively stable and indicating a strong interaction exists between the ligand and its receptor. The analysis reveals that the proteins interact with each other, with numerous amino acids involved in the interaction, mainly through hydrogen bonding. It is important to highlight that the docking scores for PEDV Nsp12, Nsp14, and Nsp16 when interacting with DNJ are −5.3 kcal/mol, −5.4 kcal/mol, and −5.6 kcal/mol, respectively, demonstrating a significant docking affinity among them. This drug specifically attaches to the active site of the protein. For example, concerning Nsp16, it forms hydrogen bonds with amino acids such as SER99, ASP98, GLY70, ASN97, CYS114, and GLY112, thereby contributing to a robust binding interaction. Additionally, hydrophobic interactions involving MET130 and ASP113 further bolster the stability of the binding. This indicates that small molecule medications could work together to hinder the virus via multiple mechanisms. Furthermore, Nsp12, Nsp14, and Nsp16 exhibit a high degree of conservation among coronaviruses, such as SARS-CoV-2 and MERS-CoV. Consequently, targeting these proteins with DNJ might be an effective strategy for suppressing various coronaviruses.

## 4. Discussion

The PEDV induces diarrhea and dehydration in nursing piglets, leading to elevated morbidity and mortality rates, thereby posing significant challenges for the swine industry. The continuous emergence of mutated strains has rendered existing PEDV vaccines often inadequate in providing effective immune protection against these new variants, complicating the management and prevention of PEDV [2,31]. Consequently, there is an urgent need to identify potential broad-spectrum antiviral agents. This study demonstrates that DNJ exhibits anti-PEDV properties by inhibiting various stages of the viral life cycle, with the most pronounced effects observed during the attachment phase. Importantly, DNJ reduces the production of ROS associated with PEDV infection, which in turn decreases MDA levels, enhances GSH-Px activity, and mitigates the inflammatory response in host cells triggered by viral entry. The findings indicate that DNJ possesses antiviral effects against PEDV and may exert its inhibitory actions by modulating signaling pathways related to oxidative stress.

Research indicates that natural products, including a variety of Chinese herbal extracts, have the potential to inhibit multiple stages of viral life cycles, thereby exhibiting antiviral properties [32]. Cepharanthine (CEP), a prominent bisbenzylisoquinoline alkaloid compound, has been shown to interfere with Porcine Deltacoronavirus (PDCoV) by targeting different phases of its life cycle, such as attachment, entry, and post-infection stages [33]. Additionally, CEP has demonstrated efficacy against human coronaviruses, including SARS-CoV and MERS-CoV, as well as porcine coronaviruses like SADS-CoV, PRRSV, and PEDV [34,35,36]. It acts as a viral infection inhibitor by modulating host signaling pathways or disrupting the viral life cycle, notably reducing the expression level of the PEDV N protein in a dose-dependent manner [37]. Furthermore, research suggests that berberine affects the replication and assembly stages of PEDV, resulting in a marked decrease in PEDV infection [38]. Several studies have emphasized that resources derived from mulberry leaves are abundant in broad-spectrum antiviral bioactive compounds [39]. DNJ is a notable alkaloid found in substantial quantities within mulberry leaves. Its antiviral properties, along with those of its derivatives, are attributed to their capacity to inhibit the assembly of the HBV pregenomic RNA capsid and disrupt the function of pregenomic RNA reverse transcriptase, thereby significantly reducing HBV gene replication and transcription [17]. Furthermore, monoribose, a derivative of DNJ, has demonstrated antiviral activity against DENV infection in primary monocyte-derived macrophages (MDMΦs) and immature dendritic cells (imDC), with effects that are dose-dependent [20]. DNJ also exhibits low toxicity in cells infected with BVDV [40]. Following DNJ administration, there was a significant reduction in the viral envelope proteins gp48 E0 and gp25 E1, impacting viral replication [10,41]. In this study, we provide the first evidence that DNJ also possesses anti-PEDV activity in Vero-E6 cells, without causing noticeable toxicity in cells, even at half of its effective concentration. The virus inactivation experiments suggest that DNJ does not directly interact with virus particles. However, during the stages of viral attachment, entry, and replication, gene expression analysis indicates that DNJ may inhibit the transcription of the PEDV-S and PEDV-N genes, resulting in varying degrees of inhibitory effects. Despite this, results from virus plaque assays demonstrate that the introduction of DNJ has minimal impact on the release phase of the virus.

Molecular docking serves as a theoretical framework for drug design by predicting ligand–target interactions. Initially, docking analysis predicted a binding interaction between tomatine and PEDV 3CLPro. Subsequent experiments confirmed that tomatine exhibits inhibitory properties against several viruses, including porcine transmissible gastroenteritis virus (TGEV), porcine reproductive and respiratory syndrome virus (PRRSV), encephalomyocarditis virus, and porcine Seneca virus [42,43,44]. Furthermore, molecular docking studies suggest that quercetin may impede the replication of PEDV by binding to the active site and pocket of the PEDV 3C-like protease [45]. The anti-PEDV properties of quercetin were corroborated using the fluorescence resonance energy transfer (FRET) technique [46]. Given the strong binding affinity of DNJ for both PEDV S1 and 3CLPro, it is hypothesized that DNJ may exhibit antiviral activity through its interactions with these targets. Furthermore, several researchers have indicated that Remdesivir acts upon the RNA-dependent RNA polymerase (RdRp) and shows efficacy against a range of RNA viruses, such as the Ebola virus and coronaviruses [47]. Our findings reveal that DNJ demonstrates a consistent binding affinity with non-structural protein 12 as well as non-structural proteins 14 and 16 of the Porcine Epidemic Diarrhea Virus (PEDV). While additional experiments are necessary for validation, these results offer a theoretical foundation for the advancement of natural compounds as broad-spectrum agents against coronaviruses.

Research suggests that viral invasion triggers the production of reactive oxygen species (ROS) [48]. We propose that the antiviral effects of DNJ are associated with the attenuation of ROS, thereby supporting cellular homeostasis. Our experiments demonstrated that PEDV infection led to increased ROS production in Vero cells, whereas DNJ effectively alleviated this oxidative stress. Notably, higher concentrations of DNJ were correlated with a reduction in ROS levels, as evidenced by decreased fluorescence. Furthermore, analysis of oxidative stress markers, such as MDA and GSH-Px, confirmed that PEDV infection disrupts cellular equilibrium. Concurrently, investigations into inflammatory markers revealed that DNJ can downregulate the inflammatory response triggered by PEDV infection, resulting in reduced MDA levels and enhanced GSH-Px activity, thereby protecting cells from oxidative damage.

This study demonstrates that DNJ can effectively inhibit the replication of PEDV in Vero-E6 cells; however, further experiments are required to verify the binding between DNJ and the predicted target. Additionally, it is important to consider that its oral bioavailability may limit its in vivo application. Furthermore, the high mutation rate of the virus could result in conformational changes in the target protein (such as polymerase or protease), thereby reducing the drug binding efficiency [49]. These represent substantial challenges that DNJ must confront when transitioning to clinical use. Nevertheless, natural products are known for their high safety and biocompatibility in drug development. Despite the aforementioned limitations, this study highlights the critical role of the natural product DNJ in combating PEDV infection and suggests its potential as a broad-spectrum antiviral drug, thereby providing a theoretical foundation for the development of new anti-PEDV strategies.

## 5. Conclusions

In conclusion, this study demonstrates that DNJ exhibits significant antiviral properties against PEDV in Vero-E6 cells, particularly impacting the virus’s attachment and replication phases. It is hypothesized that DNJ exerts its effects by alleviating oxidative stress associated with PEDV infection. These findings suggest that DNJ holds promise as a candidate for the prevention and treatment of PEDV and other porcine enteric coronavirus infections; however, further in vivo studies are necessary for comprehensive validation.

## Figures and Tables

**Table 1:** Primers used in this study.

Primer	Sequence (5′–3′)
PEDV-N-F	AAACCACGCAGCAGCAGAATG
PEDV-N-R	TGTATTTTTTCCGCTGTTGTC
PEDV-S-F	GAACTGCCATTCAGCGTATT
PEDV-S-R	ACCGAACTCAGGGTAACCAA
PEDV-M-F	TTGTATGGTGTCAAGATGGC
PEDV-M-R	AAGGATGCTGAAAGCGAAAA
PEDV-ORF3-F	TTTGCACTGTTTAAAGCGTCT
PEDV-ORF3-R	AGTAAAAGCAGACTAAACAAAGCCT
β-GAPDH-F	AACGGATTTGGTCGTATTGGG
β-GAPDH-R	CAGCTTTGGGGCAGCACTCA
IL-1β-F	GCGGCAACGAGGATGACTT
IL-1β-R	TGGCTACAACAACTGACACGG
IL-8-F	GGAACCATCTCGCTCTGTGTAA
IL-8-R	GGTCCACTCTCAATCACTCTCAG
MCP-F	ACTTGCTGCTGGTGATTCTTCT
MCP-R	CTTCTGTGCCTGCTGCTCATA
TNF-α-F	CACCACGCTCTTCTGTCT
TNF-α-R	AGATGATCTGACTGCCTGAG

## Data Availability

The data that support the findings of this study are available from the corresponding author upon reasonable request.

## Supplementary Materials

The following supporting information can be downloaded at: https://www.mdpi.com/article/10.3390/ani15091207/s1, Figure S1: The antiviral activity of DNJ at different time PEDV post-infection (the original Western blot analysis of Figure 2B); Figure S2: The antiviral activity of DNJ at different concentrations (the original Western blot analysis of Figure 2C); Figure S3: The antiviral activity of DNJ under different treatment methods (the original Western blot analysis of Figure 3B); Figure S4: The result of DNJ directly targets virus experiment (the original Western blot analysis of Figure 3C).
