# Supplementary figures and images for: Antiviral Activity of 1-Deoxynojirimycin Extracts of Mulberry Leaves Against Porcine Epidemic Diarrhea Virus
Source: Animals (Basel). 2025 Apr 23;15(9):1207. doi: 10.3390/ani15091207 (PMC12071020; doi:10.3390/ani15091207)

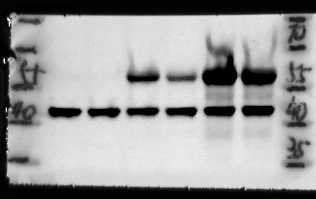

Supplement: Supplementary file 1 [file animals-15-01207-s001.zip › animals-3512138 WB original data/Figure S1/Fig.2 S1 original data.tif]

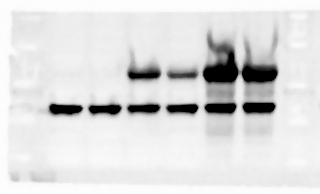

Supplement: Supplementary file 1 [file animals-15-01207-s001.zip › animals-3512138 WB original data/Figure S1/Fig.2 S1.tif]

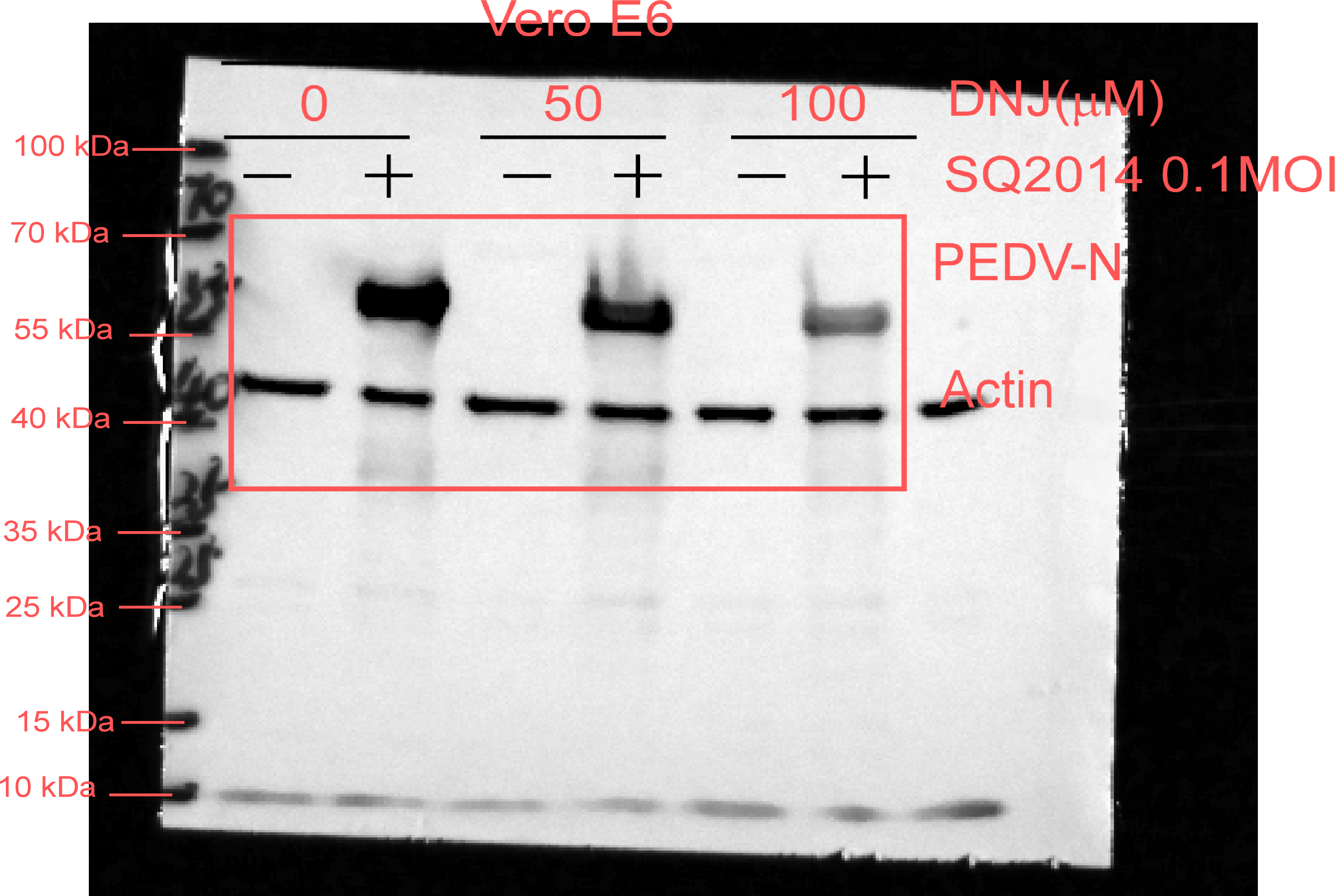

Supplement: Supplementary file 1 [file animals-15-01207-s001.zip › animals-3512138 WB original data/Figure S2/Fig.2 S2 marked.tif]

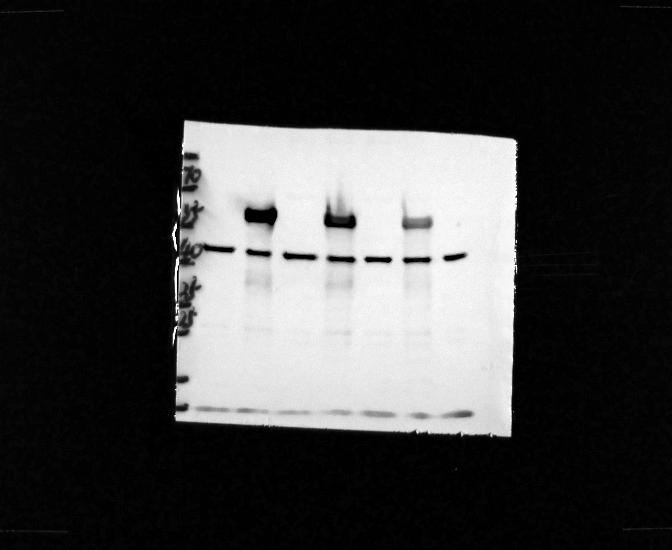

Supplement: Supplementary file 1 [file animals-15-01207-s001.zip › animals-3512138 WB original data/Figure S2/Fig.2 S2 original data.tif]

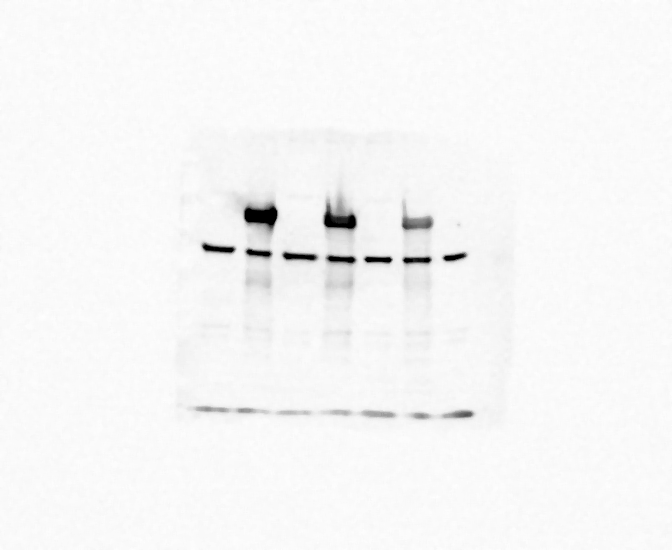

Supplement: Supplementary file 1 [file animals-15-01207-s001.zip › animals-3512138 WB original data/Figure S2/Fig.2 S2.tif]

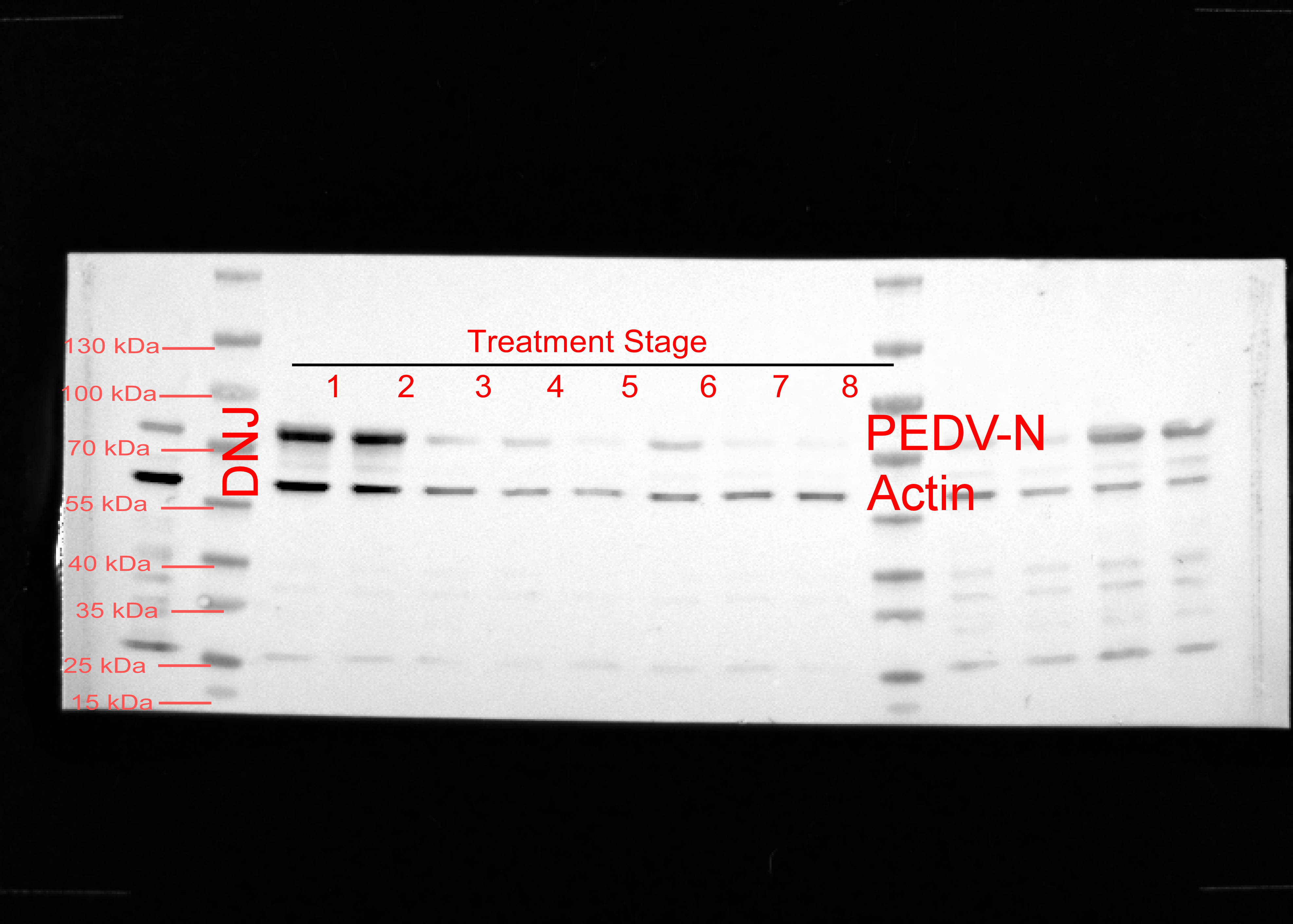

Supplement: Supplementary file 1 [file animals-15-01207-s001.zip › animals-3512138 WB original data/Figure S3/Fig.3 S3 marked.tif]

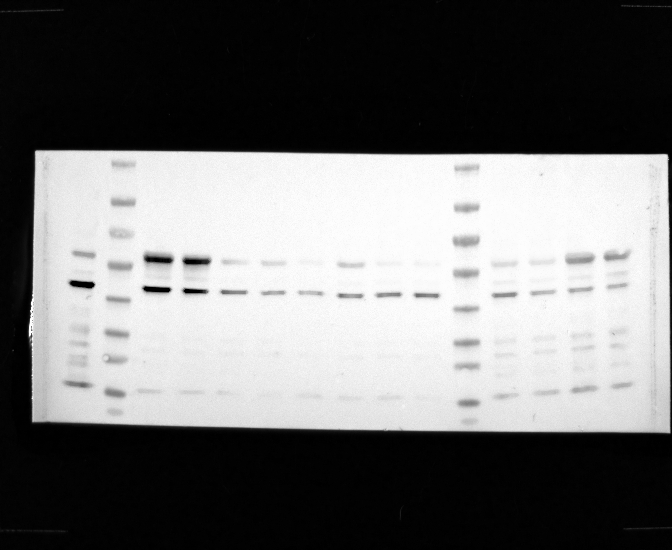

Supplement: Supplementary file 1 [file animals-15-01207-s001.zip › animals-3512138 WB original data/Figure S3/Fig.3 S3 original data.tif]

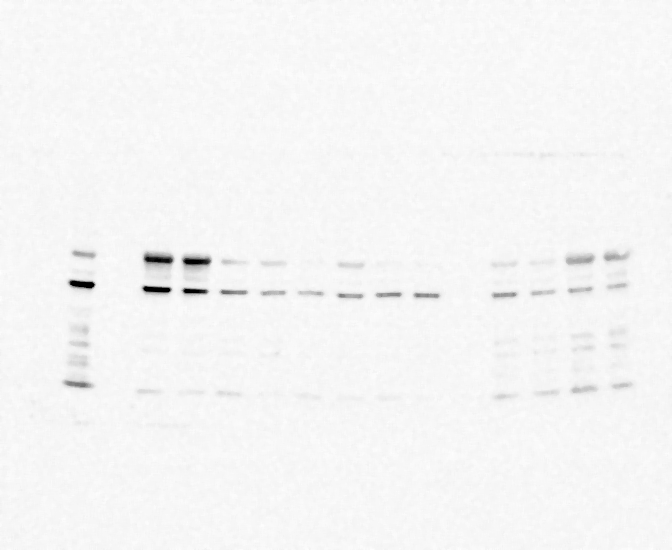

Supplement: Supplementary file 1 [file animals-15-01207-s001.zip › animals-3512138 WB original data/Figure S3/Fig.3 S3.tif]

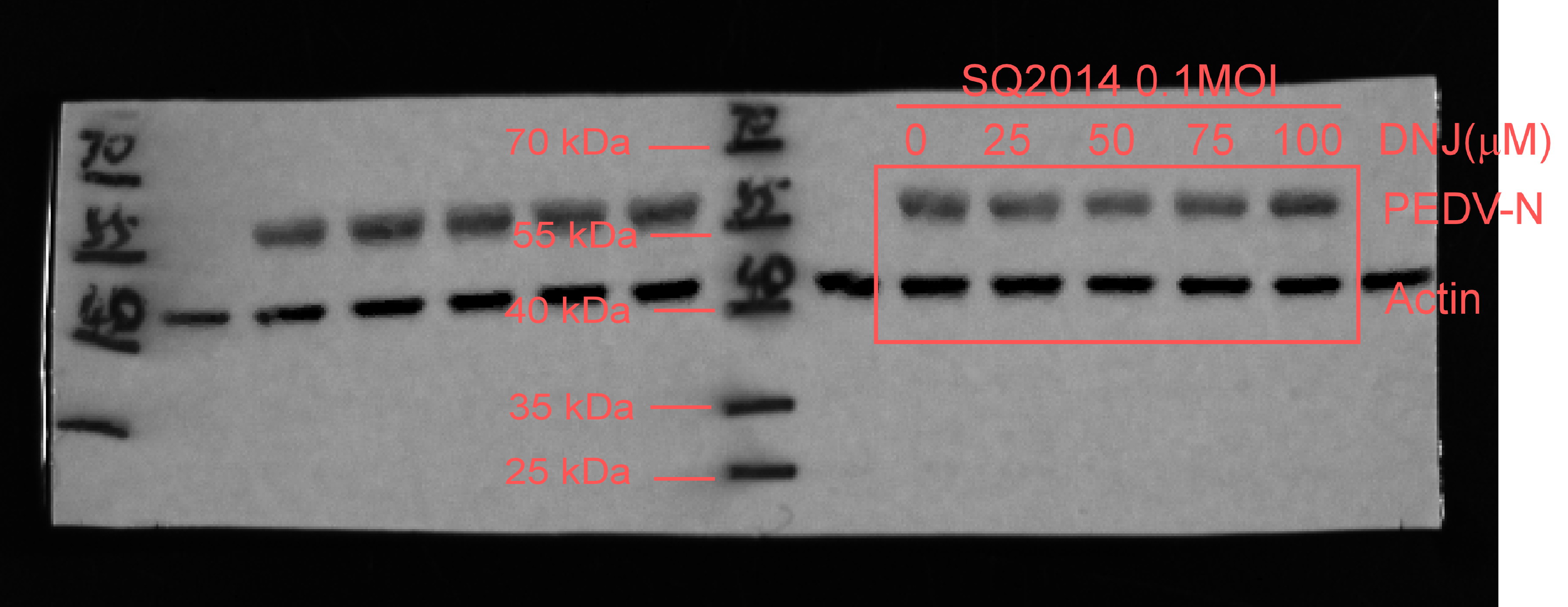

Supplement: Supplementary file 1 [file animals-15-01207-s001.zip › animals-3512138 WB original data/Figure S4/Fig.3 S4 marked.jpg]

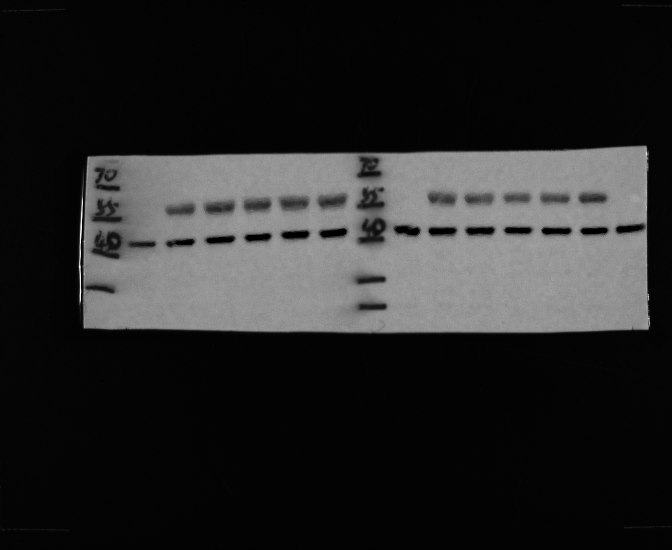

Supplement: Supplementary file 1 [file animals-15-01207-s001.zip › animals-3512138 WB original data/Figure S4/Fig.3 S4 original data.tif]

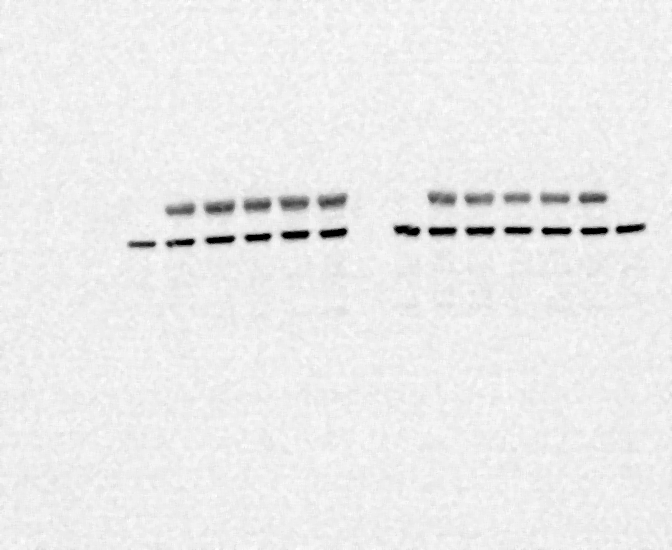

Supplement: Supplementary file 1 [file animals-15-01207-s001.zip › animals-3512138 WB original data/Figure S4/Fig.3 S4.tif]
